# Antioxidants of* Phyllanthus emblica* L. Bark Extract Provide Hepatoprotection against Ethanol-Induced Hepatic Damage: A Comparison with Silymarin

**DOI:** 10.1155/2017/3876040

**Published:** 2017-01-12

**Authors:** Renuka Chaphalkar, Kishori G. Apte, Yogesh Talekar, Shreesh Kumar Ojha, Mukesh Nandave

**Affiliations:** ^1^SPP School of Pharmacy & Technology Management, SVKM's NMIMS, Vile Parle (W), Mumbai 400056, India; ^2^National Toxicology Centre, Vadgaon Khurd, Pune 411041, India; ^3^Department of Pharmacology and Therapeutics, College of Medicine and Health Sciences, United Arab Emirates University, Al Ain, UAE

## Abstract

*Phyllanthus emblica* L. (amla) has been used in Ayurveda as a potent rasayan for treatment of hepatic disorders. Most of the pharmacological studies, however, are largely focused on PE fruit, while the rest of the parts of PE, particularly, bark, remain underinvestigated. Therefore, we aimed to investigate the protective effect of the hydroalcoholic extract of* Phyllanthus emblica* bark (PEE) in ethanol-induced hepatotoxicity model in rats. Total phenolic, flavonoid, and tannin content and in vitro antioxidant activities were determined by using H_2_O_2_ scavenging and ABTS decolorization assays. Our results showed that PEE was rich in total phenols (99.523 ± 1.91 mg GAE/g), total flavonoids (389.33 ± 1.25 mg quercetin hydrate/g), and total tannins (310 ± 0.21 mg catechin/g), which clearly support its strong antioxidant potential. HPTLC-based quantitative analysis revealed the presence of the potent antioxidants gallic acid (25.05 mg/g) and ellagic acid (13.31 mg/g). Moreover, one-month PEE treatment (500 and 1000 mg/kg, p.o.) followed by 30-day 70% ethanol (10 mL/kg) administration showed hepatoprotection as evidenced by significant restoration of ALT (*p* < 0.01), AST (*p* < 0.001), ALP (*p* < 0.05), and TP (*p* < 0.001) and further confirmed by liver histopathology. PEE-mediated hepatoprotection could be due to its free radical scavenging and antioxidant activity that may be ascribed to its antioxidant components, namely, ellagic acid and gallic acid. Thus, the results of the present study support the therapeutic claims made in Ayurveda about* Phyllanthus emblica*.

## 1. Introduction

Alcohol consumption is very common in most cultures which could be one of the reasons for alcohol abuse. Additionally, alcoholic liver disease (ALD) represents a spectrum of clinical illness and morphological changes that range from fatty liver to hepatic inflammation to progressive fibrosis and ultimately cirrhosis. Well accepted mechanisms for ethanol-induced liver injury are fat accumulation in hepatocytes, CYP2E1 induction, and oxidative stress-mediated hepatocyte damage [1]. CYP2E1, the main variant of cytochrome P450 enzymes and also a component of the microsomal ethanol oxidizing system (MEOS), plays an important role in ethanol metabolism. Following ethanol consumption, CYP2E1 activity can increase up to fourfold [2]. Alcohol dehydrogenase- and MEOS-mediated ethanol metabolism leads to production of acetaldehyde, which generates ROS. The resultant ROS production causes oxidative stress, endoplasmic reticulum stress, and steatosis. Another source of ROS production is through activation of NADPH oxidase in hepatocytes. ROS-mediated oxidative stress is considered as the most important causative factor in the pathology of ALD. The ROS contribute to liver damage through a variety of mechanisms including inactivation of antioxidant enzymes, depletion of reduced glutathione, alteration of the breakdown of fat molecules, and lipid peroxidation. Collectively all these alterations lead to inflammation and apoptosis in hepatocytes [3]. On the other hand, the cellular antioxidant milieu gets depleted; particularly, chronic consumption of ethanol leads to reduced glutathione depletion, which makes hepatocytes more sensitive to ROS-mediated oxidative stress [4].

Herbal extracts exhibit protective mechanism against oxidative stress by enhancing antioxidant enzyme activities and averting GSH depletion [5].* Phyllanthus emblica* L. (PE) is a tropical and subtropical tree that belongs to the family Euphorbiaceae and is distributed throughout the Deccan, coastal districts, Kashmir, and deciduous forests of Madhya Pradesh in India. PE is also widely distributed in most tropical and subtropical countries including China and Indonesia and in the Malay Peninsula. PE is native to tropical southeastern Asia, particularly, in central and southern India, Nepal, Pakistan, Bangladesh, Bhutan, Sri Lanka, Myanmar, and the Mascarene Islands [6]. PE is a medium to large deciduous plant 7 to 19 meters in height growing wild or cultivated throughout tropical India with a grey bark and reddish wood. The bark of PE appears to be shiny greyish brown or greyish green and thick up to 12 mm. According to the two main classic texts on Ayurveda, Charak Samhita, and Sushruta Samhita, amla is regarded as “the best among rejuvenating herbs and sour fruits.” Various beneficial effects of* emblica* have been described in “Materia Medica,” a classical Indian text on the Ayurvedic system of medicine [7]. PE is a major ingredient in many Ayurvedic preparations including Triphala and Chyawanprash, a general tonic for people of all ages for overall mental and physical well-being. Traditionally PE has been used in Ayurveda for the treatment of diarrhoea and fever, as a diuretic, in inflammation, skin sores, and wounds, and as a potent rasayan in hepatic disorders [8, 9]. The fruit of the plant has been prescribed for different pharmacological activities like antioxidant [10], antitumor [11], gastroprotective [12], antitussive [13], hepatoprotective [14], and antidiabetic [15].

Entire parts of PE, including fruit, flower, seed, leaf, root, and bark, have been widely used in various folk systems, such as traditional system of Indian medicine (Ayurveda), traditional Chinese medicine, Tibetan medicine, and Arab medicine (Unani). The minorities living in the southwest of China use the root for the treatment of eczema and fruits for the treatment of jaundice and diarrhoea, whereas, in the Nepal, it is used as an astringent and hemostatic [16–18]. Bark of PE possesses strong antioxidant and radical scavenging activities demonstrating that it can be correlated to the presence of polyphenols [19]. Being a rich source of tannin (21–33%), the bark of PE is one of the main raw materials used for tannin extract production in China. Most of the pharmacological studies, however, are largely focused on PE fruit, while the rest of the parts of PE, particularly, bark, remain underinvestigated. Currently only wound healing activity of the bark of PE has been reported [20].

As PE contains antioxidant constituents like total phenols, flavonoids, and tannins in abundance and ROS-mediated oxidative stress is considered as major mechanism responsible for alcohol-induced liver damage, we intended to evaluate the hepatoprotective activity of hydroalcoholic bark extract of* P. emblica *(PEE) in rats intoxicated with ethanol.

## 2. Materials and Methods

### 2.1. Chemicals

Silymarin was purchased from Microlabs, Bangalore, India. Gallic acid and ellagic acid were purchased from Natural Remedies Pvt. Ltd., Bangalore, India. Diagnostic kits for assaying ALT, AST, ALP, and total protein were purchased from Crest Biosystems, Goa, India.

### 2.2. Plant Collection and Authentication

Fresh bark of PE was collected from Kem Village, Solapur District of Maharashtra, India, during monsoon season, washed thoroughly with water, and shade-dried. A voucher specimen (No: RMC-1.BSI/WRC/Tech.2012) was identified and authenticated by Botanical Survey of India, Pune.

### 2.3. Extraction

Powdered dried bark (100 g) was extracted with 250 mL mixture of absolute ethanol and water in the ratio of 7 : 3 using a Soxhlet apparatus. Further, hydroalcoholic extract was evaporated to dryness by distillation under reduced pressure in rotatory evaporator. The yield of PEE was 3 g per 100 g of PE bark. The PEE was then subjected to characterization and phytochemical screening.

### 2.4. Phytochemical Screening

PEE was subjected to phytochemical evaluation to examine the presence of carbohydrate, flavonoids, tannins, glycosides, saponins, alkaloids, sterols and triterpenoids, and amino acids. Total phenols, flavonoids, and tannins were also quantified in PEE.

#### 2.4.1. Total Phenols

Total phenolic content in PEE was determined based on Folin-Ciocalteu (FC) colorimetric method [21]. In brief, 1 mg/mL of the PEE was prepared and mixed with FC reagent. After 3 min, 3 mL of sodium carbonate (20%) was added to the reaction mixture and allowed to stand for 2 h with occasional shaking. The absorbance of the blue colour was measured at 760 nm using spectrophotometer. Total phenolic content was calculated from the calibration curve of gallic acid and concentration of total phenols expressed as gallic acid equivalent in mg/g of dry PEE.

#### 2.4.2. Flavonoids

Total flavonoid content was determined using the aluminium chloride assay through colorimetric assay method [22]. One millilitre of PEE was diluted with 2 mL of distilled water and, after 5 min, 3 mL of 5% sodium nitrite and 0.3 mL of 10% aluminium trichloride was added. After 6 min, 2 mL of 1 M sodium hydroxide was added and volume was made up to 10 mL with distilled water. After incubation a red coloured complex was formed which was measured at 510 nm. Flavonol content was calculated from the calibration curve of quercetin and was expressed as quercetin equivalent in mg/g of PEE.

#### 2.4.3. Tannins

Total tannin content was determined using modified vanillin assay [23, 24]. PEE (1 mL of 1 mg/mL) was incubated in water bath for a brief period to bring it to temperature equilibrium. The working vanillin reagent (5 mL) was added to the sample and 5 mL 4% HCl was used as blank. Both sample and blank were incubated for 20 min and absorbance was read at 500 nm. Total tannin content was calculated from the calibration curve of catechin and was expressed as catechin equivalents mg/g of PEE.

### 2.5. Characterization of PEE Using HPTLC Analysis

#### 2.5.1. Instrumentation and Chromatographic Conditions

PEE was standardized using HPTLC analysis in accordance with Jeganathan and Kannan [25] using a solution of the PEE (20 mg/mL) and standard stock solutions of gallic acid and ellagic acid stock (10 *μ*g/mL), prepared in HPLC grade methanol. The stock solutions and PEE were filtered through 0.45 *μ* syringe filter and then subjected to HPTLC analysis. The analysis was performed by comparing and interpolating the PEE peak area with that of the standard gallic acid and ellagic acid from the calibration curve.

HPTLC aluminium plates precoated with silica gel F60_254_ (10 × 10 cm) with 200 *μ*m thickness (Merck) were used as the stationary phase. The mobile phase used was toluene : ethyl acetate : formic acid (2 : 7 : 2). Ascending development was carried out in a twin trough glass chamber saturated with the mobile phase until the solvent reached a maximum front distance of 160 mm. The plate was saturated for 30 minutes at room temperature (25 ± 2°C) and subsequently allowed to dry at room temperature. The separated bands on the HPTLC plates were scanned over the wavelength of 200–400 nm with a maximum absorbance at wavelength of 280 nm. The peak areas were recorded to obtain the concentrations of gallic acid and ellagic acid.

### 2.6. In Vitro Antioxidant Activity

#### 2.6.1. Hydrogen Peroxide Scavenging Activity

Briefly, various concentrations of PEE were prepared and mixed with 0.3 mL of 4 mM solution prepared in phosphate buffer (0.1 M, pH 7.4) and incubated for 10 min. The absorbance of the solution was taken at 230 nm against blank solution containing the PEE without H_2_O_2_. Ascorbic acid was used as a standard. All measurements were made in duplicate and average of these two observations was considered. The scavenging effect was then calculated according to the following equation:

(1) $$\begin{array}{c} H_{2}O_{2}\;\;scavenging\;\;activity=1-\left(\;\frac{A_{s}}{A_{c}}\;\right)\times 100, \end{array}$$

where *A*_*c*_ = absorbance of control and *A*_*s*_ = absorbance of extract.

The concentration equivalent to ascorbic acid was calculated by plotting the values of the PEE on standard curve of ascorbic acid. EC_50_ value (mg/mL) is the concentration at which the scavenging activity was 50% [26].

#### 2.6.2. ABTS Radical Scavenging Activity

In this method, ABTS was dissolved in distilled water to achieve concentration of 7 mM. ABTS radical cation (ABTS^•+^) was produced by reacting ABTS stock solution with 2.45 mM of potassium persulfate and the mixture was allowed to stand in the dark at room temperature for 12–16 h before use. The percent scavenging activity of the PEE was determined by calculating the % inhibition by the following formula and results were compared with ascorbic acid as standard:

(2) $$\begin{array}{c} {ABTS}^{•+}\;\;scavenging\;\left(\;\%\;\right)=1-\left(\;\frac{A_{s}}{A_{c}}\;\right)\times 100, \end{array}$$

where *A*_*c*_ = absorbance of control and *A*_*s*_ = absorbance of the PEE.

The concentration equivalent to ascorbic acid was calculated by plotting the values of the PEE on standard curve of ascorbic acid [27].

## 3. Hepatoprotective Activity

### 3.1. Animals

Male Wistar rats (150–200 g) were kept at National Toxicology Centre (Pune, India). Animals were housed in group of six (three of parallel sex in one cage) in polypropylene cages and acclimatized to standard laboratory conditions (temperature 25 ± 10°C, relative humidity 50 ± 15%) one week prior to the actual commencement of the experiment. Light-dark cycle of 12-12 h was maintained for animals. They were provided with standard food pellets (NAV Maharashtra Chakan Oil Mills Ltd., Pune) and tap water ad libitum. The study and protocol were approved by Institutional Animal Ethical Committee (CPCSEA/IAEC/NTC/P-200/2012).

#### 3.1.1. Ethanol-Induced Hepatotoxicity

Animals were randomized into five different groups containing six animals in each group. Group I served as normal control and was given 1 mL/kg saline p.o. for 30 days. Group II served as disease control and received 70% ethanol (10 mL/kg p.o.) for 30 days. Group III served as positive control and was given silymarin (25 mg/kg p.o.) followed by 70% ethanol (10 mL/kg p.o.) for 30 days. Groups IV and V served as treatment groups and received PEE oral dose of 500 and 1000 mg/kg, respectively, followed by 70% ethanol (10 mL/kg p.o.) for 30 days.

#### 3.1.2. Biochemical Estimation

At the end of 4 weeks, animals were starved overnight and then sacrificed by overdose of anesthesia. Blood was collected and kept for 1 h at room temperature for clotting. Serum was separated by centrifugation at 3000 rpm for 20 mins and the biochemical parameters serum alanine aminotransferase (ALT), aspartate aminotransferase (AST), alkaline phosphatase (ALP) activities, and total protein content were determined by spectrophotometric procedures, using the ERBA assay kits (ERBA Diagnostics Mannheim GmbH, Mannheim, Germany).

#### 3.1.3. Histopathological Examination

The animals used in the study were sacrificed and liver tissue was examined grossly and weighed. A small portion of liver tissue of each animal was fixed in 10% neutral buffered formalin processed. Then it was embedded in paraffin wax to obtain 5-6 *μ*m thick hematoxylin and eosin stained sections for examination [28].

Five fields were viewed by blinding for histopathological signs at a magnification of 200. Sinusoidal congestion (in hepatic parenchyma), degenerative changes of hepatocytes, cellular swelling, vacuolar changes with granular cytoplasm of hepatocytes, necrotic changes of hepatic parenchyma, loss of nucleus, fragmentation of nuclei, centrilobular, midzonal necrosis, bile duct hyperplasia, perivascular lymphoid aggregation, infiltration of mononuclear cells (MNC), formation of microgranuloma, fatty liver (lipid deposition in hepatocyte), and steatosis were graded as the following: no abnormality detected (NAD), minimal pathological changes (+), mild pathological changes (++), moderate pathological changes (+++), and severe pathological changes (++++).

### 3.2. Acute Toxicity Study

The acute toxicity study for PEE was performed using male albino mice. The animals were fasted overnight prior to the experiment and maintained under standard conditions. The study was conducted as per Organization of Economic Cooperation and Development (OECD) Test guidelines 425 on Acute Oral Toxicity [29]. The study was carried out in a stepwise procedure. In step I, three animals were used and given 2000 mg/kg of the PEE. When mortality was found to be unlikely, step II was carried out. In step II, additional three animals were again given the PEE at a dose of 2000 mg/kg and observed for 14 days.

### 3.3. Statistical Analysis

Data obtained from in vitro experiments was expressed as mean ± SEM. For in vivo experiments and statistical differences between the treatments and the control were evaluated by One Way ANOVA followed by Dunnett's test. A probability value of *p* < 0.05 was considered as significant.

## 4. Results

### 4.1. Qualitative Analysis of Phytochemicals Present in PEE

As shown in Table 1, qualitative analysis of PEE revealed the presence of flavonoids, tannins, glycosides, saponins, and alkaloids.

### 4.2. Quantification of Total Phenolic, Flavonoids, and Tannin Contents Present in PEE

As shown in Table 2, the quantitative phytochemical screening of PEE revealed the presence of total phenols, flavonoids, and tannins.

#### 4.2.1. Total Phenolic Content

The total phenolic content of the PEE was determined from the regression equation of calibration curve (*R*^2^ = 0.996) and found to be 99.523 mg/g which was equivalent to 275 *μ*g/mL of gallic acid.

#### 4.2.2. Total Flavonoid Content

The total flavonoid content of the PEE was determined from the regression equation of calibration curve (*R*^2^ = 0.996) and found to be 389.33 mg/g which was equivalent to 200 *μ*g/mL of quercetin hydrate.

#### 4.2.3. Total Tannin Content

The total tannin content of the PEE was determined from the regression equation of calibration curve (*R*^2^ = 0.993) and found to be 310 mg/g which was equivalent to 300 *μ*g/mL of catechin.

### 4.3. HPTLC Analysis of Gallic Acid and Ellagic Acid Present in PEE

Linearity of the calibration curves of gallic acid and ellagic acid was tested by linear regression analysis and found to be linear in the concentration range 10–60 *μ*g/mL with good correlation coefficient (*r*^2^) of more than 0.99. The peaks of gallic acid and ellagic acid in the PEE were identified by comparing retention time of reference gallic acid and ellagic acid. The amount of gallic acid and ellagic acid in the PEE was estimated to be about 25.05 mg/g of gallic acid and 13.31 mg/g of ellagic acid.

### 4.4. In Vitro Antioxidant Activity

#### 4.4.1. Hydrogen Peroxide Scavenging Activity

The ability of PEE and ascorbic acid to scavenge hydrogen peroxide radicals is shown in Table 3. PEE was capable of scavenging free radicals in dose dependent manner. Up to concentration of 200 *μ*g/mL, the percentage inhibition of PEE (43.20%) was almost comparable to that of ascorbic acid (55.39%). However, at 250 *μ*g/mL of PEE, the percentage inhibition of PEE was 79.62%, which was found to be better than ascorbic acid (71.34%). The IC_50_ value of PEE was 188.80 *μ*g/mL while that of ascorbic acid was 177.7 *μ*g/mL.

#### 4.4.2. ABTS Radical Scavenging Activity

The ABTS radical scavenging activity of PEE was found to be concentration dependent. The maximum inhibition of ABTS radical at the concentration of 250 *μ*g/mL was 42.91% which was less effective than that of standard (Table 4). The IC_50_ value of the PEE was 329.20 *μ*g/mL while that of ascorbic acid was 133.96 *μ*g/mL.

### 4.5. Biochemical Estimation

As shown in Table 5, the ethanol intoxicated group showed significant increase in serum levels of ALT (*p* < 0.001), AST (*p* < 0.001), and ALP (*p* < 0.05) along with decrease in the total protein (*p* < 0.001) compared to normal rats, indicating hepatotoxic effect of ethanol. After administration of PEE at the dose of 500 mg/kg, a statistically significant decrease in the elevated levels of ALT (*p* < 0.01), AST (*p* < 0.001), and ALP (*p* < 0.05) was observed along with improvement in TP content (*p* < 0.001) towards normal. Administration of 1000 mg/kg dose of PEE also restored elevated levels of ALT (*p* < 0.05), AST (*p* < 0.01), and TP (*p* < 0.01) but the effect of ALP was not statistically significant. Effect of PEE at dose of 500 mg/kg on ALT, AST, ALP, and TP content was comparable to that of silymarin, a standard hepatoprotective agent. When tested and standard drug treatment groups were compared with normal control group, there was no significant difference for all the mentioned biochemical parameters. Difference between test and standard drug treatment groups was also statistically nonsignificant.

### 4.6. Histopathological Examination

**Figure 1:**
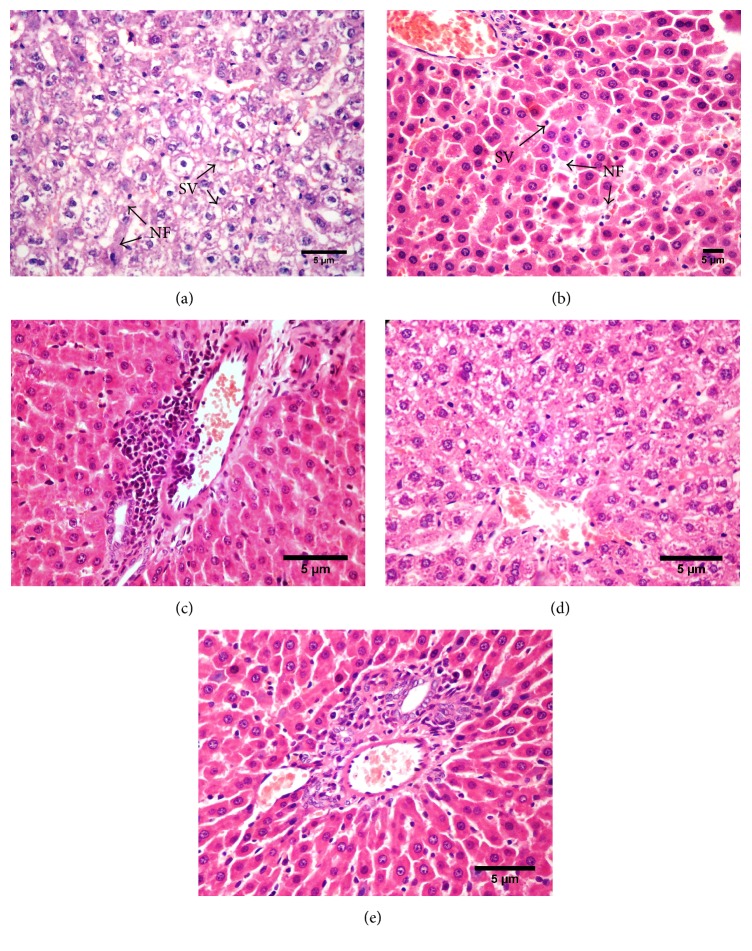
Photomicrographs of liver sections stained with hematoxylin and eosin. (a and b) Ethanol treated rat liver, (c) PEE500 mg/kg + ethanol treated, (d) PEE1000 mg/kg + ethanol treated, and (e) silymarin 25 mg/kg + ethanol treated (200x) [SV: degenerated hepatocytes with cellular swelling and vacuolar changes in cytoplasm; NF: necrotic foci with loss of nucleus of hepatocytes].

Histology of the liver sections of rats of different groups is shown in Figure 1. Examination of the histological activity index (HAI, Table 6) of the liver found that animals of normal control group showed normal histoarchitecture of hepatic parenchyma with hepatocytes arranged in cord like fashion around the central vein. The nucleus and cytoplasm showed normal histological features with intactness and normal morphology of cytoplasm and blood vessels and bile duct in portal triad.

The liver sections from disease control group showed moderate degree of damage to hepatic parenchyma with diffuse cellular swelling, degenerative changes in cytoplasm, and nucleus and focal areas of necrosis in the midzonal area. Many hepatocytes showed accumulation of lipoid material while few areas showed derangement of hepatic cords and necrotic changes of hepatocytes with occasional basophilia of nucleus and vacuolar cytoplasmic changes in hepatocytes. Focal areas of haemorrhages in the degenerative foci of hepatic parenchyma were also seen in a few sections. The overall features indicated a moderate degree of pathological changes leading to condition of fatty liver with degenerative and ensuing necrobiotic changes (Figures 1(a) and 1(b)).

Liver sections from animals of PEE 500 mg/kg group showed minimal to mild degree of histolopathological changes in the hepatic parenchyma. The hepatocytes showed minimal pathomorphological features of hepatocytes with multifocal areas of cellular swelling, vacuolar changes, and focal infiltration of mononuclear cells in hepatic parenchyma. Focal hyperplasia of bile duct in portal triad was also noted in one animal of this group with mild pathological changes in hepatic parenchyma.

As compared to PEE 500 mg/kg group, the histological evaluation from liver sections of PEE 1000 mg/kg group showed only minimal changes in hepatic parenchyma with normal hepatocytes and blood vessels and bile ducts. Focal changes of minimal degree were seen in only a few hepatocytes with less severity without any necrotic features.

The liver sections of SIL 25 mg/kg group showed normal hepatic parenchyma with normal hepatocytes and blood vessels and bile ducts. Focal changes of minimal degree were seen in only a few hepatocytes with less severity without any necrotic features. The observations of SIL 25, PEE 500, and PEE 1000 mg/kg groups were comparable with each other with slight differences.

### 4.7. Acute Toxicity Study

PEE did not show any significant change in body weight and behavioural pattern. There were no signs and symptoms of toxicity or mortality up to the dose level of 2000 mg/kg (data not shown). The LD_50_ was found to be greater than 2000 mg/kg with a cutoff at 5000 mg/kg.

## 5. Discussion

Ethanol is a familiar hepatotoxic chemical used for inducing liver damage in animals. Toxicity produced by ethanol is related to its oxidative metabolism by enzymes like alcohol dehydrogenase and CYP2E1 [30]. The metabolism of ethanol results in elevated production of superoxide [31] and hydrogen peroxide free radicals [32, 33]. Additionally, an increased level of acetaldehyde also decreases hepatic glutathione content and impairs the defence system of body that neutralizes free radicals and activates phagocytic cells. This overproduction of free radicals ultimately results in an increased level of lipid peroxidation followed by formation of adducts with cellular proteins and nucleic acids. These adducts eventually limit the function of hepatocytes [33].

In the present study, the hepatoprotective effect of PEE was evaluated using alcohol-induced hepatotoxicity model, since it is clinically relevant. Ethanol produces a group of characteristic effects in the liver leading to ALD [34]. In a similar manner, we also found biochemical and architectural perturbations in liver of the ethanol intoxicated rats. Chronic administration of ethanol led to a significant elevation of serum levels of ALT, AST, and ALP. The rise in the ALT level is usually accompanied by an elevation in the levels of AST, which plays a role in the conversion of amino acids to keto acids. Decrease in the levels of total protein (TP) was observed in the alcohol treated rats indicating the destruction in the number of hepatic cells, which may result in a decrease in hepatic capacity to synthesize protein.

Alcohol intoxication-mediated oxidative stress causes peroxidation of cell membrane lipids and alters membrane phospholipid composition and fluidity, thereby increasing the cell membrane permeability. Such an injured cell membrane causes leakage of various enzymes including ALT, AST, and ALT into blood circulation as shown by abnormally high levels of serum hepatic markers in disease control group. The present observations are in line with the previous studies which indicated that ALT, AST, and ALP are normally located in the cytoplasm and released into systemic circulation after hepatic cellular injury [35]. An elevated serum ALP level could be due to defective hepatic excretion or by increased production of ALP by hepatic parenchymal or duct cells. Ethanol-mediated hepatocyte injury is also evidenced in our study where chronic administration of ethanol resulted in accumulation of lipoid material in hepatocytes, derangement of hepatic cords, and necrotic and vacuolar cytoplasmic changes in hepatocytes.

However, oral administration of PEE significantly decreased serum levels of AST, ALP, and ALT at both doses. Compared to 1000 mg/kg dose of PEE, its 500 mg/kg dose was found to be more effective. The hepatoprotective effect observed with PEE at the dose of 1000 mg/kg was not significantly prominent compared to that observed with PEE 500 mg/kg. Obtained hepatoprotection with PEE could be due to restoration of hepatocyte membrane integrity by PEE, which led to the restoration of hepatic enzymes. Additionally, suppression of elevated ALP activities with concurrent increase in the total protein content suggests the stability of biliary dysfunction in rat liver during hepatic injuries with toxicants. Treatment with PEE markedly elevated the total protein level which was comparable with standard drug silymarin. Hepatoprotective activity of PEE was found to be more prominent at the dose of 500 mg/kg than that at 1000 mg/kg compared to control group.

Apart from ethanol-mediated oxidative stress, alcohol is a well-known genotoxic substance that causes genomic instability. This could be another mechanism that contributes to alcohol-induced liver damage. A recent work by Guo and Wang has demonstrated that PE could activate spindle assembly checkpoint and prevent mitotic aberrations and genomic instability in human cells. Therefore, the hepatoprotective potential of PE may also be attributed to the reinforcement of endogenous mechanisms against alcohol-induced genomic instability [36].

Preliminary phytochemical screening of PEE as well as quantification of the PEE revealed the presence of tannins, flavonoids, and phenols. Additionally, quantitative characterization of PEE by HPTLC method revealed it to be a rich source of ellagic acid and gallic acid. All these measured phytoconstituents in the present study have been reported earlier for their antioxidant potential by many researchers [37–39]. In this context, an antioxidant and hepatoprotective activity of PEE observed in this study could be attributed to its high content of gallic acid and ellagic acid. A large body of evidence shows the hepatoprotective potential of gallic acid and ellagic acid [40–44].

In the present study, results of in vitro antioxidant assays like hydrogen peroxide assay (IC_50_ value: 188.80 *μ*g/mL) and ABTS assay (IC_50_ value: 329.20 *μ*g/mL) noticeably showed dose dependent free radical scavenging potential of PEE. Our study results are in line with the findings of previous studies [45, 46]. As both in vitro methods are widely used because of their relevance to biological systems, in vitro free radical scavenging effect of PEE correlates to its in vivo antioxidant activity. Studies have shown that PE attenuates oxidative stress and related damage by increasing the endogenous antioxidant enzymes. Very recently, Tahir et al. reported that PE leaves extract ameliorates pulmonary fibrosis by elevating the activities of catalase, superoxide dismutase, glutathione peroxidase, and reduced glutathione in the pulmonary samples of rat [47]. Moreover, reports suggest that PE increases the antioxidant enzymes and prevents ethanol-induced toxic effects [48]. Considering the findings of previous studies, the hepatoprotective effect of PEE observed in the present study could be due to restoration and/or increase in the endogenous antioxidant milieu.

Although, silymarin, an isolated hepatoprotective flavonoid, is one of the most studied phytoconstituents in animals as well as humans, PE has many advantages over silymarin. PE is an enriched source of ascorbic acid and other phytoconstituents including linoleic acid, emblicanin A and emblicanin B, gallic acids, chebulic acid, ellagic acid, quercetin, and rutin. Synergistic interactions or multifactorial effects between these phytoconstituents make PE a better hepatoprotective herbal modality. Briefly, phytoconstituents present in PE work in a holistic manner. Although the dose response relationship can be easily established while using silymarin (a single isolated constituent), complete characterization of the PEE can address the limitation of the whole extract of PE. In this study, the hepatoprotective effect of silymarin was marginally better than that of PE as evidenced by biochemical and histopathological parameters. Silymarin and PE share many mechanisms that are responsible for their therapeutic effect including free radical scavenging activity, antioxidant activity, augmentation of endogenous antioxidant enzymes, attenuation of lipid peroxidation and inflammation, and stimulation of protein synthesis.

In summary, the in vitro and in vivo studies confirmed the antioxidant as well as hepatoprotective potential of hydroalcoholic extract of* P. emblica* bark and found it to be comparable with silymarin, a standard hepatoprotective phytoconstituent.

## 6. Conclusion

The data of the present study demonstrate that PEE possesses potent antioxidant activity against free radicals and provides significant protection against alcohol-induced liver damage. Biochemical analysis showed that PEE efficiently restores the levels of ALT, AST, ALP, and TP, which is further supported by histopathological observations. The resulting antioxidant and hepatoprotective activities of the PEE can be attributed to the presence of gallic acid, ellagic acid, polyphenols, and flavonoids. Although the findings corroborate the therapeutic potential of PE bark, further studies are needed to identify and isolate the polyphenolic compounds responsible for the antioxidant activity of PEE. Further investigation on PEE can then decide its use in patients with alcoholic liver disease.

## Figures and Tables

**Table 1:** Qualitative analysis of phytochemicals present in PEE.

Phytochemical analyzed	Test method	Inference
Carbohydrate	Molisch's test	−
	Fehling's test	−
Flavonoids	Alkaline reagent test	**+**
	Ferric chloride test	**++**
Tannins	Ferric chloride test	**++**
	Potassium dichromate test	**+++**
Glycosides	Keller-Kiliani's test	**+**
Saponins	Foam test	**+++**
Alkaloids	Mayer's test	**+**
	Dragendorff's test	**+**
Sterols and triterpenoids	Salkowski test	−
Amino acids	Ninhydrin test	−

(+++): appreciable amount; (++) moderate amount; (+) trace amount; (−) completely absent.

**Table 2:** The total phenolic, flavonoids, and tannin contents present in PEE.

Parameters	Hydroalcoholic extract of PE bark
Total phenolic content (mg of GAE/g of PEE)	99.523 ± 1.91
Total flavonoid content (mg of quercetin hydrate/g of PEE)	389.33 ± 1.25
Total tannin content (mg of catechin/g of PEE)	310 ± 0.21

Values are expressed as mean ± SEM of three replicates.

**Table 3:** H_2_O_2_ radical scavenging activity of PEE (*n* = 6).

Concentration [*µ*g/ml]	% scavenging activity of ascorbic acid	% scavenging activity of PEE
10	6.82 ± 1.106	7.85 ± 2.427
50	14.63 ± 0.571	14.02 ± 5.603
100	28.80 ± 0.7495	23.91 ± 2.998
150	40.82 ± 1.999	32.01 ± 2.248
200	55.39 ± 0.2855	43.20 ± 3.301
250	71.34 ± 0.2498	79.62 ± 2.541

Values are expressed as a mean ± SEM.

**Table 4:** ABTS radical scavenging activity of PEE (*n* = 6).

Concentration [*µ*g/ml]	% inhibition	Concentration equivalent to ascorbic acid [*µ*g/ml]
50	21.79 ± 0.011	34.0
100	27.52 ± 0.001	55.0
150	31.74 ± 0.011	70.5
200	36.23 ± 0.007	87
250	42.91 ± 0.002	111.5

Values are expressed as mean ± SEM.

**Table 5:** Effect of PEE on ALT, AST, ALP, and TP in ethanol-induced liver damage in rats.

Study groups	Treatment administered	ALT	AST	ALP	TP
Normal control	Saline (1 mL/kg/day)	49.49 ± 4.647	155.4 ± 6.197	270.71 ± 48.66	5.2 ± 0.31
Disease control	70% ethanol (10 mL/kg/day)	103.9 ± 16.33^###^	301.8 ± 17.91^###^	396.4 ± 17.07^#^	3.017 ± 0.31^###^
SIL25	Silymarin (25 mg/kg/day)	56.20 ± 1.953^*∗∗*^	230.5 ± 21.65^*∗*^	278.6 ± 4.351^*∗*^	4.9 ± 0.50^*∗∗*^
PEE500	PEE (500 mg/kg/day)	61.20 ± 4.693^*∗∗*^	189.8 ± 8.84^*∗∗∗*^	267.1 ± 14.15^*∗*^	5.283 ± 0.25^*∗∗∗*^
PEE1000	PEE (1000 mg/kg/day)	69.03 ± 3.66^*∗*^	210.8 ± 17.82^*∗∗*^	360.9 ± 44.92	5.133 ± 0.35^*∗∗*^

ALT: alanine aminotransferase, AST: aspartate aminotransferase, ALP: alkaline phosphatase, and TP: total protein. All values are expressed as mean ± SEM of at least 6 animals from each experimental group. Data found significant when One Way ANOVA followed by Dunnett's multiple comparison test performed. *p* < 0.05 was considered to be significant. Statistical significance (^#^*p* < 0.05; ^##^*p* < 0.01; ^###^*p* < 0.001) compared with the normal control group and (^*∗*^*p* < 0.05; ^*∗∗*^*p* < 0.01; ^*∗∗∗*^*p* < 0.001) compared with the disease control group.

**Table 6:** Effect of PEE on histopathological activity index in ethanol-induced liver damage in rats.

Study groups	Sinusoidal congestion in hepatic parenchyma	Degenerative changes of hepatocytes, cellular swelling, vacuolar changes with granular cytoplasm of hepatocytes	Necrotic changes of hepatic parenchyma, loss of nucleus, fragmentation of nuclei, centrilobular, midzonal necrosis	Bile duct hyperplasia	Perivascular lymphoid aggregation	Infiltration of mononuclear cells (MNC), formation of microgranuloma	Fatty liver (lipid deposition in hepatocyte), steatosis	Overall pathological grade (lesion score)
Normal control	+/focal	NAD	NAD	NAD	+/focal	NAD	+/focal	NAD
Disease control	++	+ ++	++	+	+	NAD	++	+++
SIL25	NAD	+/focal	NAD	NAD	NAD	NAD	NAD	+
PEE500	NAD	+/focal	+/focal	+	NAD	+/focal	NAD	++
PEE1000	NAD	+	+	+/focal	NAD	NAD	NAD	+

*n* = 4 per experimental group. No abnormality detected (NAD), minimal pathological changes (+), mild pathological changes (++), moderate pathological changes (+++), and severe pathological changes (++++). Focal and minimal changes may not be significant for alteration of functional capacity of the organ.
